# Dataset on evaluation of intra-specific genetic diversity and phylogenetic relationship of *Launaea taraxacifolia* (Willd.) Amin ex C. Jeffrey in southwest Nigeria using rbcL gene maker

**DOI:** 10.1016/j.dib.2024.110531

**Published:** 2024-05-13

**Authors:** Abiodun Sunday Oyelakin, Jacob Olagbenro Popoola, Favour Olanrewaju Babalola, Taiwo Adebowale Bamiro, Gloria Oladapo, Victor Olamide Oluwatuyi, Oluwatobi Faith Oke, Al-fuad Sobayo

**Affiliations:** a Department of Pure and Applied Botany, College of Biosciences, Federal University of Agriculture, Abeokuta, P.M. B. 2240, Abeokuta, Ogun State, Nigeria; b Pure and Applied Biology programme, College of Agriculture, Engineering and Science, Bowen University, Iwo, Osun State, Nigeria

**Keywords:** *Launaea taraxacifolia*, Genetic diversity, Improvement, Breeding, Conservation

## Abstract

*Launaea taraxacifolia* (Willd.) Amin Ex C. Jeffrey (Asteraceae) popularly known as “African wild lettuce” is a neglected, underutilized, and sometimes classified as a weed in West and Central Africa. The plant has been naturalized in numerous regions of the world, including Asia, North America, Europe, and North Africa. This highly nutritional and medicinal leafy vegetable is endemic to some states in southwest, Nigeria. People who utilize the species still depend largely on its spontaneous appearance in the wild, except for some herbalists who cultivate it for therapeutic uses. Its domestication and cultivation are still at infant stage. Without the intervention of breeders, the full potential of this species would remain untapped. The inadequate information about the genetic diversity of *L. taraxacifolia* hinders its improvement through breeding programme and for conservation purposes, hence this dataset. A total of fifteen (15) accessions of L. *taraxacifolia* were collected from Oyo, Osun and Ogun states in Nigeria. The accessions were partitioned into three populations according to their collection states and subjected to DNA extraction, polymerase chain reaction amplification and Sanger sequencing using ribulose-1,5-carboxylase/oxygenase large subunit gene (rbcL). The dataset composed of partial rbcL gene sequences which provides information on *L. taraxacifolia* distribution in southwest, Nigeria, its genetic diversity, single nucleotide polymorphic information, codon usage bias and amino acids molecular weight profile. The dataset recorded a relatively low number of segregating sites (3), total number of haplotypes (4), and nucleotide diversity (0.298) with a high gene diversity (0.667) and average number of nucleotide differences (0.895). A significant low level of genetic differentiation (Fst) was recorded for the population in the decreasing order of 0.103 (Ogun and Oyo populations), 0.000 (Ogun and Osun populations) and −0.222 (Oyo and Osun populations). The unweighted pair group method with arithmetic mean revealed the genetic diversity and phylogenetic relationships of *L. taraxacifolia* accessions which could be explored for its domestication, cultivation, genetic improvement and conservation in Nigeria.

Specifications Table

**Table:** 

Subject	Biological Sciences
Specific subject area	Botany, Agricultural, Genetic diversity, Plant breeding, and Conservation
Data types	Tables, Figure
How data were acquired	Collection of *Launaea taraxacifolia* root cuttings from open fields and wild spaces, PCR amplification of rbcL gene, and DNA Sanger Sequencing.
Data format	Raw, and Analysed
Description collected data	The root cuttings of 15 accessions of *Launaea taraxacifolia* were collected from three states where the species is endemic in southwest, Nigeria, namely Oyo, Ogun and Osun States. The accessions were categorized into three populations according to their endemic states (Oyo: five accessions; Ogun: six accessions; and Osun: four accessions). The plants were identified as *Launaea taraxacifolia* at the Federal University of Agriculture, Abeokuta (FUNAAB) Herbarium, Nigeria. The root cuttings were planted in single lines per accession in 5 replicates in the screen house for two weeks. Genomic DNA was extracted from young leaves and subjected to polymerase chain reaction (PCR) and sanger sequencing using the ribulose-1,5-carboxylase/oxygenase large subunit (rbcL) gene marker. The nucleotide and amino acid compositions, sequence length, number of invariable sites/monomorphic, genetic differentiation and codon usage bias were analysed using DnaSP 6.0. The codon usage indices and amino acid residues with their molecular weight profiles were estimated using CodonW. The phylogenetic relationships among the accessions were inferred using the UPGMA method.
Data source location	The locations are presented in Table 1.
Data accessibility	The accessions sequence data have been deposited in NCBI GenBank and the following accession numbers were assigned; OR785724.1, OR785725.1, OR785726.1, OR785727.1, OR785728.1, OR785729.1, OR785730.1, OR785731.1, OR785732.1, OR785733.1, OR785734.1, OR785735.1, OR785736.1, OR785737.1, OR785738.1. Popset accession number: OR785724 Direct URL to data: https://www.ncbi.nlm.nih.gov/popset/?term=OR785724

## 1 Value of the Data

• The dataset including sequence relatedness, number of polymorphic sites, total number of mutations, haplotype (gene) diversity, codon usage bias, and amino acids weight profile provide insight into the genetic relatedness and phylogenetic relationships among the fifteen accessions of *Launaea taraxacifolia* studied from Nigeria.
• It highlighted the availability of *L. taraxacifolia* within the studied areas valuable for possible germplasm collection for cultivation, conservation, and breeding purposes.
• The relatively low genetic differentiation among the three populations of *L. taraxacifolia* indicates a high commonality in the nucleotide contents and information which may possibly corroborate the endemism of the species to the southwest, Nigeria.
• The dataset underscores the potential of InDel haplotype genetic information to initiate mutation breeding to enlarge the existing narrow gene pool of *L. taraxacifolia*.
• The amino acid profiles generated from *L. taraxacifolia* could be employed to promote its nutritional values to improve its local and regional utilisation for food, nutrition and medicine as well as for its infra-generic delimitation in Nigeria.
• The genetic diversity and phylogenetic relationships can benefit breeders and scientists to facilitate *L. taraxacifolia* domestication, genetic improvement, and commercial cultivation in Nigeria.

## 2 Objectives

The research was initiated to provide information on location and distribution, assess intraspecific genetic diversity and phylogenetic relationships among fifteen accessions of *L. taraxacifolia* collected from three states in southwest Nigeria where the species is endemic using partial rbcL gene sequence.

## 3 Data Description

**Fig. 1:**
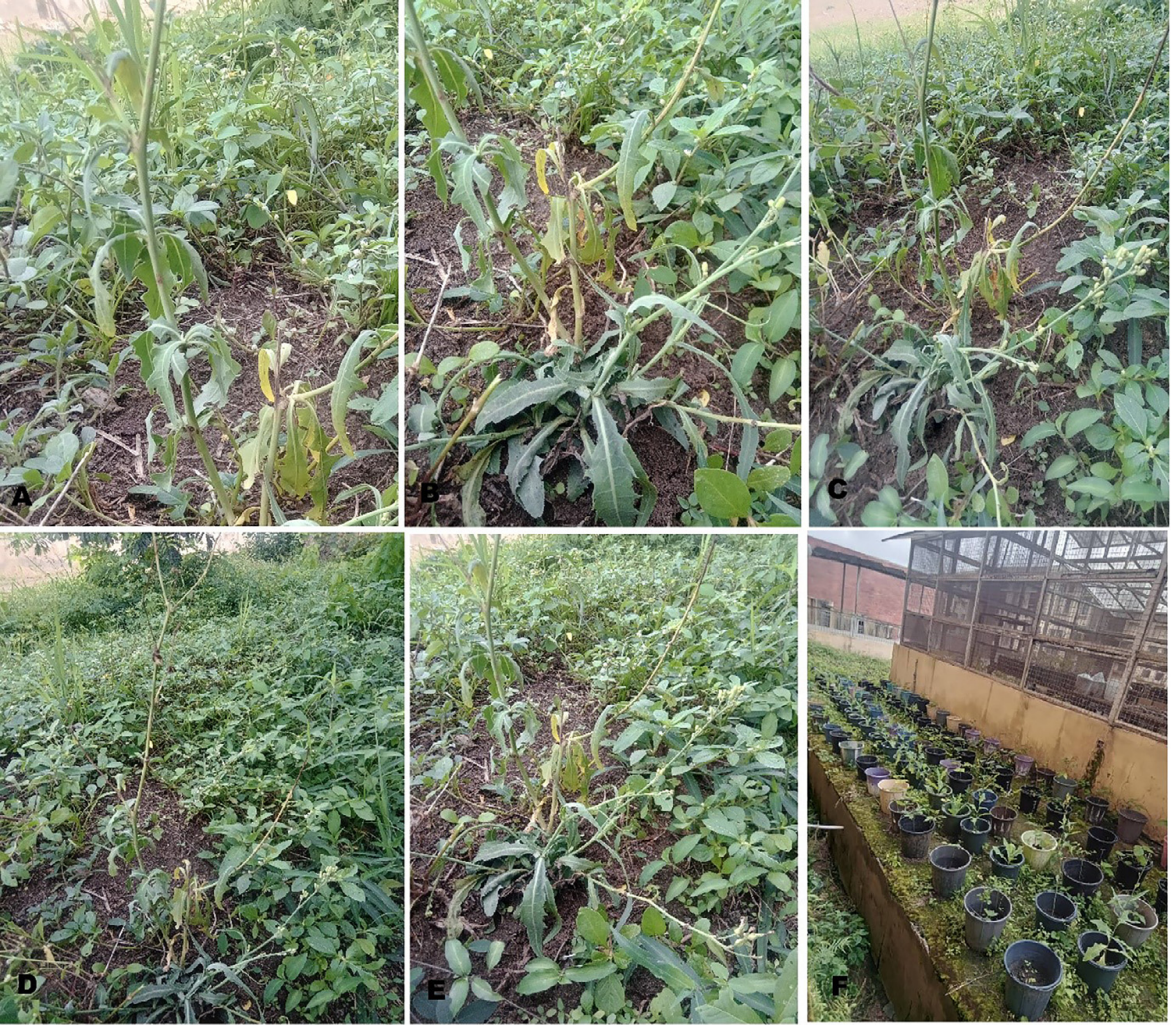
Images of *L. taraxacifolia* collected from the wild and plants raised from the root cuttings. A. OgLt001 B. OgLt006 C. OyLt008 D. OyLt009 E. OsLt012 F. Accessions raised from root cuttings

**Fig. 2:**
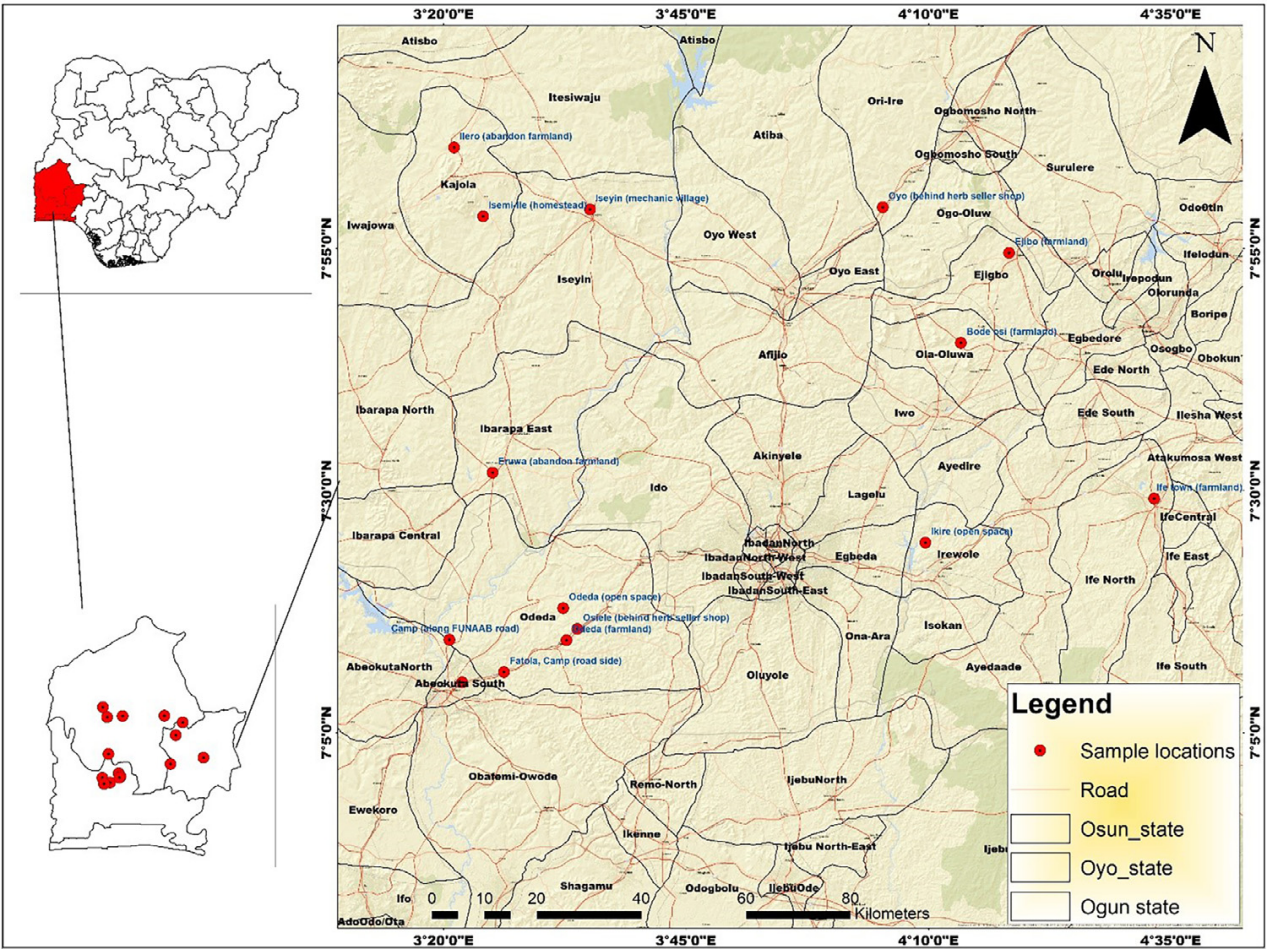
Mapping of collection sites of the *Launaea taraxacifolia* accessions from Osun, Oyo and Ogun States, Nigeria.

**Fig. 3:**
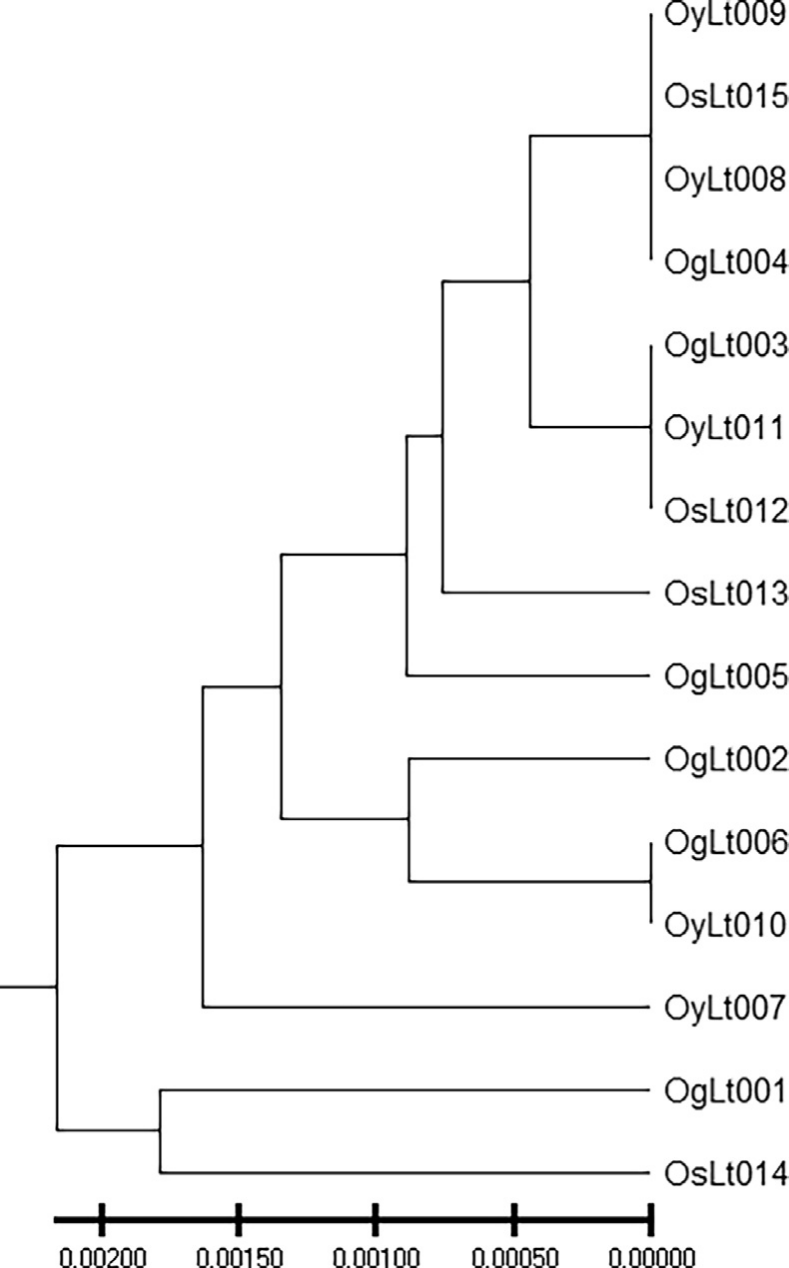
Phylogenetic relationships among *L. taraxacifolia* accessions using rbcL gene sequence.

*Launaea taraxacifolia* is a perennial West Africa tropical plant commonly known as African wild lettuce in the Asteraceae family. It is called 'Efo yanrin' by Yoruba people in the southwestern part of Nigeria. It thrives on waste dumping places, and abandoned farmlands. *L. taraxacifolia* is one of the important neglected and underutilized leafy vegetables in Nigeria [1,2]. It is used to increase milk production in cattle and induce multiple births in livestock [[2], [3], [4]]. Despite the nutritional and medicinal values of *L. taraxacifolia*, it remains uncultivated and largely harvested from the wild. It is imperative to genetically improve this species, save it from impending extinction and promote its domestication and cultivation. The Rbcl sequencing information on *L. taraxacifolia* could provide insights into its genetic relatedness and facilitate its genetic improvement via marker-assisted strategies. The phenotypic images of *L. taraxacifolia* accessions growth habit collected from the wild and as planted in the screen house is shown in Fig. 1. The field collection details including passport data, location coordinates, altitude of *L. taraxacifolia* accessions and Genbank accession numbers for the deposited sequences are described in Table 1. Fig. 2 describes the map of the collection sites. The Genbank accession numbers, percentage identity, matched organism, and sequence length (bp) are presented in Table 2. The percentage nucleotide contents involving % GC, % T(U), %(C), % A and % G of the 15 accessions of *L. taraxacifolia* are described in Table 3. The description of genetic diversity parameters such as polymorphic and monomorphic sites, parsimony information sites, total number of mutations, number of haplotypes, and nucleotide diversity is recorded in Table 4. Single nucleotide polymorphisms among the accessions is presented in Table 5 while genetic differentiation among the three populations is recorded in Table 6. The codon bias usage information, frequencies, average codons, and relative synonymous codon usage (RSCU) for *L. taraxacifolia* studied are described in Table 7 while codon usage indices for each accession of *L. taraxacifolia* are presented in Table 8. The amino acid molecular weight profile of *L. taraxacifolia* sequences is shown in Table 9. Fig. 3 presents the evolutionary history of the species based on phylogenetic tree inferred using the UPGMA method.

**Table 1:** Genbank accession number, collection number, passport data, location coordinates and altitude of *L. taraxacifolia* accessions studied.

S/N	Genbank accession number	Collection number	Area of collection	LGA	State	Latitude NS	Longitude EW
1	OR785724.1	OgLt001	Camp (along FUNAAB road)	Odeda	Ogun	7°11′14.8″	3°26′04.9″
2	OR785725.1	OgLt002	Odeda (open space)	Odeda	Ogun	7°13′53.0″	3°31′39.0″
3	OR785726.1	OgLt003	Osiele (behind herb seller shop)	Odeda	Ogun	7°19′83.0″	3°47′00.0″
4	OR785727.1	OgLt004	Fatola, Camp (roadside)	Odeda	Ogun	7°11′07.2″	3°26′04.1″
5	OR785728.1	OgLt005	Odeda (farmland)	Odeda	Ogun	7°13′53.0″	3°31′39.0″
6	OR785729.1	OgLt006	Adatan (herb seller shop)	Abeokuta South	Ogun	7°14′52.4″	3°32′76.9″
7	OR785730.1	OyLt007	Isemi-Ile (homestead)	Kajola	Oyo	7°98′65.3″	3°37′47.9″
8	OR785731.1	OyLt008	Ilero (abandon farmland)	Kajola	Oyo	8°09′62.9″	3°35′02.3″
9	OR785732.1	OyLt009	Eruwa (abandon farmland)	Ibarapa East	Oyo	7°53′25.3″	3°42′26.9″
10	OR785733.1	OyLt010	Iseyin (mechanic village)	Iseyin	Oyo	7°96′53.5″	3°56′96.8″
11	OR785734.1	OyLt011	Oyo (behind herb seller shop)	Atiba	Oyo	7°85′61.3″	3°90′95.9″
12	OR785735.1	OsLt012	Ife town (farmland)	Ife	Osun	7°45′96.7″	4°55′69.7″
13	OR785736.1	OsLt013	Ejibo (farmland)	Ejibo	Osun	7°83′24.3″	4°37′29.1″
14	OR785737.1	OsLt014	Ikire (open space)	Irewole	Osun	7°37′75.1″	4°18′25.6″
15	OR785738.1	OsLt015	Bode osi (farmland)	Ola oluwa	Osun	7°83′24.3″	4°37′27.1″

**Table 2:** Summary of the deposited sequences, Genbank accession numbers, matched organism, and sequence length.

Sequence name	GenBank Accession Number	Matched Organism	% Identity	SL (bp)
*Launaea taraxacifolia* isolate OgLt002 ribulose-1,5-bisphosphate carboxylase/oxygenase large subunit (rbcL) gene, partial cds; chloroplast.	OR785725.1	*Launaea taraxacifolia*	100 %	549
*Launaea taraxacifolia* isolate OgLt003 ribulose-1,5-bisphosphate carboxylase/oxygenase large subunit (rbcL) gene, partial cds; chloroplast.	OR785726.1	*Launaea taraxacifolia*	100 %	549
*Launaea taraxacifolia* isolate OgLt004 ribulose-1,5-bisphosphate carboxylase/oxygenase large subunit (rbcL) gene, partial cds; chloroplast.	OR785727.1	*Launaea taraxacifolia*	100 %	549
*Launaea taraxacifolia* isolate OgLt006 ribulose-1,5-bisphosphate carboxylase/oxygenase large subunit (rbcL) gene, partial cds; chloroplast.	OR785729.1	*Launaea taraxacifolia*	100 %	549
*Launaea taraxacifolia* isolate OgLt009 ribulose-1,5-bisphosphate carboxylase/oxygenase large subunit (rbcL) gene, partial cds; chloroplast.	OR785732.1	*Launaea taraxacifolia*	100 %	549
*Launaea taraxacifolia* isolate OgLt010 ribulose-1,5-bisphosphate carboxylase/oxygenase large subunit (rbcL) gene, partial cds; chloroplast.	OR785733.1	*Launaea taraxacifolia*	100 %	549
*Launaea taraxacifolia* isolate OgLt012 ribulose-1,5-bisphosphate carboxylase/oxygenase large subunit (rbcL) gene, partial cds; chloroplast.	OR785735.1	*Launaea taraxacifolia*	100 %	549
*Launaea taraxacifolia* isolate OgLt013 ribulose-1,5-bisphosphate carboxylase/oxygenase large subunit (rbcL) gene, partial cds; chloroplast.	OR785736.1	*Launaea taraxacifolia*	100 %	549
*Launaea taraxacifolia* isolate OgLt001 ribulose-1,5-bisphosphate carboxylase/oxygenase large subunit (rbcL) gene, partial cds; chloroplast.	OR785724.1	*Launaea taraxacifolia*	100 %	549
*Launaea taraxacifolia* isolate OgLt005 ribulose-1,5-bisphosphate carboxylase/oxygenase large subunit (rbcL) gene, partial cds; chloroplast.	OR785728.1	*Launaea taraxacifolia*	100 %	548
*Launaea taraxacifolia* isolate OgLt007 ribulose-1,5-bisphosphate carboxylase/oxygenase large subunit (rbcL) gene, partial cds; chloroplast.	OR785730.1	*Launaea taraxacifolia*	100 %	548
*Launaea taraxacifolia* isolate OgLt008 ribulose-1,5-bisphosphate carboxylase/oxygenase large subunit (rbcL) gene, partial cds; chloroplast.	OR785731.1	*Launaea taraxacifolia*	100 %	548
*Launaea taraxacifolia* isolate OgLt011 ribulose-1,5-bisphosphate carboxylase/oxygenase large subunit (rbcL) gene, partial cds; chloroplast.	OR785734.1	*Launaea taraxacifolia*	100 %	548
*Launaea taraxacifolia* isolate OgLt014 ribulose-1,5-bisphosphate carboxylase/oxygenase large subunit (rbcL) gene, partial cds; chloroplast.	OR785737.1	*Launaea taraxacifolia*	100 %	548
*Launaea taraxacifolia* isolate OgLt015 ribulose-1,5-bisphosphate carboxylase/oxygenase large subunit (rbcL) gene, partial cds; chloroplast.	OR785738.1	*Launaea taraxacifolia*	100 %	548

**Table 3:** Genbank Accession number, percentage nucleotide content of the rbcL gene of *L. taraxacifolia*.

S/N	Genbank Accession No.	% GC	T(U)	%C	%A	%G
1	OR785724.1	42.81	30.55	19.36	26.64	23.45
2	OR785725.1	43.04	30.34	19.58	26.63	23.46
3	OR785726.1	42.83	30.44	19.47	26.73	23.36
4	OR785727.1	42.93	30.39	19.61	26.68	23.32
5	OR785728.1	42.81	30.55	19.54	26.64	23.27
6	OR785729.1	42.75	30.57	19.61	26.68	23.14
7	OR785730.1	43.11	30.39	19.61	26.50	23.50
8	OR785731.1	43.19	30.44	19.65	26.37	23.54
9	OR785732.1	42.76	30.59	19.50	26.65	23.26
10	OR785733.1	42.75	30.57	19.61	26.68	23.14
11	OR785734.1	42.83	30.62	19.47	26.55	23.36
12	OR785735.1	42.75	30.57	19.43	26.68	23.32
13	OR785736.1	43.01	30.44	19.47	26.55	23.54
14	OR785737.1	42.73	30.85	19.33	26.42	23.40
15	OR785738.1	43.01	30.44	19.65	26.55	23.36
	**Average**	**42.89**	**30.52**	**19.53**	**26.60**	**23.36**

**Table 4:** Genetic diversity indices among fifteen (15) landraces of *Launaea teraxaxifolia*.

Index	Value
Number of nucleotides	15
Total number of sites (excluding sites with gaps / missing data	3
Number of sites	3
Invariable (monomorphic) sites	0
Number of polymorphic (segregating sites)	3
Parsimony informative sites	2
Total number of mutations (Eta)	3
Number of haplotypes (h)	4
Haplotype (gene) diversity (Hd)	0.667
Variance of Haplotype diversity + SD	0.00982 ± 0.099
Nucleotide diversity (Pi)	0.298
Average number of nucleotide differences (k)	0.895
Total number of (InDel and non-InDel) sites analysed	3 + 0 = 3

**Table 5:** Single nucleotide polymorphic of the accessions of *L. taraxacifolia* studied.

S/N	Accessions	SN	SN	SN
1	OgLt001	G	g	A
2	OgLt002	.	.	.
3	OgLt003	.	.	.
4	OgLt004	.	.	.
5	OgLt005	.	.	G
6	OgLt006	.	.	.
7	OyLt007	.	a	G
8	OyLt008	.	.	G
9	OyLt009	A	.	.
10	OyLt010	.	.	.
11	OyLt011	.	.	G
12	OsLt012	.	.	.
13	OsLt013	.	.	.
14	OsLt014	.	a	G
15	OsLt015	.	.	G

Accessions correspond to collection numbers which also align with the Genebank accession numbers on Table 1.

**Table 6:** Genetic differentiation among the three populations of *L. taraxacifolia*.

POPULATION 1	POPULATION 2	Hs	Ks	Kxy	Gst	DeltaSt	GammaSt	Nst	Fst	Dxy	Da
Pop_1	Pop_2	0.576	0.818	0.967	0.112	0.044	0.167	0.188	0.103	0.322	0.033
Pop_1	Pop_3	0.500	0.667	0.750	−0.034	0.028	0.139	0.065	0.000	0.250	0.000
Pop_2	Pop_3	0.873	1.296	1.050	−0.069	0.009	0.025	−0.351	−0.222	0.350	−0.078

**Hs** = haplotype-based statistics; **Ks** = statistics based on nucleotide sequences, **Kxy** = average proportion of nucleotide difference between populations; **Gst** = genetic differentiation index based on the frequency of haplotypes; **GammaS**t = genetic differentiation coefficient; **Fst** = genetic differentiation; **Dxy** = average number of nucleotide substitutions per site between populations; **Da** = net nucleotide substitutions per site between populations. Pop_1 = Ogun State population, Pop_2 = Oyo State population, and Pop_3 = Osun State population

**Table 7:** Codon usage bias: Relative Synonymous Codon Usage (RSCU) and count for *L. taraxacifolia* rbcL nucleotides.

Codon	Count	RSCU	Codon	Count	RSCU	Codon	Count	RSCU	Codon	Count	RSCU
UUU(F)	6	1.5	UCU(S)	3	2.57	UAU(Y)	9	1.38	UGU(C)	2	1.33
UUC(F)	2	0.5	UCC(S)	2	1.71	UAC(Y)	4	0.62	UGC(C)	1	0.67
UUA(L)	2	0.71	UCA(S)	0	0	UAA(*)	0.1	3	UGA(*)	0	0
UUG(L)	6	2.12	UCG(S)	0	0	UAG(*)	0	0	UGG(W)	2	1
CUU(L)	5	1.76	CCU(P)	9	2.77	CAU(H)	0.2	0.33	CGU(R)	5	3
CUC(L)	0	0	CCC(P)	1	0.31	CAC(H)	1	1.67	CGC(R)	0	0
CUA(L)	1	0.35	CCA(P)	1	0.31	CAA(Q)	4.7	2	CGA(R)	3	1.8
CUG(L)	3	1.06	CCG(P)	2	0.62	CAG(Q)	0	0	CGG(R)	0	0
AUU(I)	5	2.14	ACU(T)	11	2.75	AAU(N)	1	0.5	AGU(S)	1	0.86
AUC(I)	2	0.86	ACC(T)	2	0.5	AAC(N)	3	1.5	AGC(S)	1	0.86
AUA(I)	0	0	ACA(T)	2	0.5	AAA(K)	8.1	1.6	AGA(R)	2	1.2
AUG(M)	1	1	ACG(T)	1	0.25	AAG(K)	2	0.4	AGG(R)	0	0
GUU(V)	7	2.15	GCU(A)	7	1.86	GAU(D)	7.6	1.58	GGU(G)	8.1	1.67
GUC(V)	0	0	GCC(A)	3	0.8	GAC(D)	2	0.42	GGC(G)	1	0.21
GUA(V)	5	1.54	GCA(A)	4	1.06	GAA(E)	10	1.67	GGA(G)	6.1	1.27
GUG(V)	1	0.31	GCG(A)	1.1	0.28	GAG(E)	2	0.33	GGG(G)	4.1	0.84
Average# codons=186

**Table 8:** Codon usage Indices for each of the 15*L. taraxacifolia* rbcL sequences.

Accessions	T3s	C3s	A3s	G3s	CAI	CBI	Fop	Nc	GC3s	GC	L_sym	L_aa	Aromo
OR785724.1	0.32	0.34	0.33	0.24	0.15	−0.05	0.39	47.30	0.47	0.46	155.00	168.00	0.18
OR785725.1	0.30	0.25	0.30	0.40	0.16	−0.04	0.34	61.00	0.51	0.44	170.00	182.00	0.08
OR785726.1	0.54	0.16	0.35	0.16	0.26	0.06	0.45	45.12	0.26	0.43	183.00	187.00	0.12
OR785727.1	0.31	0.34	0.32	0.25	0.15	−0.06	0.39	46.91	0.48	0.46	156.00	169.00	0.18
OR785728.1	0.29	0.25	0.30	0.39	0.15	−0.03	0.34	61.00	0.51	0.43	168.00	180.00	0.08
OR785729.1	0.31	0.35	0.33	0.24	0.15	−0.05	0.39	46.81	0.48	0.46	156.00	169.00	0.18
OR785730.1	0.31	0.34	0.33	0.25	0.15	−0.05	0.39	46.06	0.47	0.46	156.00	170.00	0.17
OR785731.1	0.32	0.34	0.33	0.25	0.15	−0.05	0.39	48.01	0.47	0.46	156.00	169.00	0.18
OR785732.1	0.56	0.15	0.34	0.16	0.28	0.09	0.47	44.18	0.25	0.43	183.00	186.00	0.12
OR785733.1	0.31	0.35	0.33	0.24	0.15	−0.05	0.39	46.81	0.48	0.46	156.00	169.00	0.18
OR785734.1	0.31	0.34	0.33	0.24	0.15	−0.05	0.39	46.74	0.48	0.46	155.00	169.00	0.18
OR785735.1	0.31	0.34	0.33	0.24	0.15	−0.05	0.39	46.74	0.48	0.46	155.00	169.00	0.18
OR785736.1	0.54	0.16	0.35	0.17	0.26	0.06	0.45	45.20	0.26	0.43	183.00	187.00	0.12
OR785737.1	0.30	0.25	0.30	0.39	0.16	−0.04	0.34	61.00	0.51	0.43	168.00	180.00	0.08
OR785738.1	0.31	0.34	0.32	0.25	0.15	−0.06	0.39	46.91	0.48	0.46	156.00	169.00	0.18

***CAI*** - *Codon* Adaptation Index, ***CBI*** - *Codon* Bias Index, **FoP** - Frequency of optimal codons, **Aromo** - Aromaticity.

**Table 9:** Amino Acid molecular weight profile of the *L. taraxacifolia* sequences.

Gene name	Amino acids																				
	Phe	Leu	Ile	Met	Val	Ser	Pro	Thr	Ala	Tyr	TER	His	Gln	Asn	Lys	Asp	Glu	Cys	Trp	Arg	Gly
OR785724.1	8	12	9	4	8	26	8	5	6	13	19	3	4	6	3	3	3	12	9	19	7
OR785725.1	7	44	10	8	16	6	8	9	4	3	7	4	6	8	15	4	7	5	4	6	8
OR785726.1	8	17	7	1	12	9	13	17	14	12	1	1	4	4	10	10	12	4	3	10	19
OR785727.1	8	12	10	4	8	26	8	5	6	13	19	3	4	6	3	3	3	12	9	20	6
OR785728.1	7	44	10	8	16	7	8	9	4	3	7	3	4	8	15	4	7	5	4	7	7
OR785729.1	8	12	10	4	8	26	8	5	6	13	19	3	4	6	3	3	3	13	9	19	6
OR785730.1	7	13	11	4	8	26	8	5	7	12	18	3	4	6	3	3	3	12	10	19	6
OR785731.1	8	12	9	4	8	26	8	5	7	13	19	3	5	6	2	4	3	12	9	19	6
OR785732.1	8	17	7	1	13	7	13	17	15	12	0	1	4	4	11	10	12	3	2	10	19
OR785733.1	8	12	10	4	8	26	8	5	6	13	19	3	4	6	3	3	3	13	9	19	6
OR785734.1	8	12	10	4	8	26	8	5	6	13	19	3	4	6	3	3	3	12	10	19	6
OR785735.1	8	12	10	4	8	26	8	5	6	13	19	3	4	6	3	3	3	12	10	19	6
OR785736.1	8	17	7	1	12	9	13	17	14	12	1	1	4	4	10	10	12	4	3	10	19
OR785737.1	8	44	9	8	16	6	8	10	4	3	8	3	4	8	15	5	7	5	4	6	7
OR785738.1	8	12	10	4	8	26	8	5	6	13	19	3	4	6	3	3	3	12	9	20	6

## 4 Experimental Design, Materials and Methods

### 4.1 Plant material

Fifteen (15) accessions of *L. taraxacifolia* were collected from three states in southwest, Nigeria namely Oyo, Ogun and Osun. They were assigned collection numbers at the point of collection (Table 1). The plants were identified as *L. taraxacifolia* in the Herbarium of the Department of Pure and Applied Botany (PAB) at the Federal University of Agriculture, Abeokuta (FUNAAB), Nigeria where voucher specimens were deposited. The root cuttings were transplanted into perforated 5-l buckets filled with topsoil and arranged in the PAB screen house. The buckets were irrigated once every two days with 5 l of tap water and monitored for leaf emergence. Young leaf samples collected from each accession were put into separate Ziploc bags, and transported to the laboratory of Inqaba Biotec West Africa Ltd, Ibadan, Nigeria for genomic DNA extraction and molecular studies.

### 4.2 Genomic DNA extraction

The genomic DNA was extracted from the sample leaves following the procedures described by Popoola et al. [5].

### 4.3 PCR amplification, visualization, and purification

Amplification of the PCR reactions for the rbcL regions was done using the following composition: 12.5 µL of NEB OneTaq 2X Master Mix with Standard Buffer (Catalogue No. M0482S), 2 µL of genomic DNA (10–30 ng/µl), 0.5 µL RbclA_F (5-ATGTCACCACAAACAGAGACTAAAGC-3 (Tm = 53 °C); and 724R RbclA_R (5-GTAAAATCAAGTCCACCCG-3 (Tm = 53 °C) (10 µM), and 9.5 µL of nuclease-free water (Catalogue No. E476) to make up a final volume of 25 µL. The PCR amplicons were sequenced at Inqaba Biotec West Africa Ltd Ibadan, Nigeria using Zymo Research DNA Sequencing Clean-Up Kits protocol according to manufacturer's instructions and as described by Popoola et al. [5].

### 4.4 Data analysis

The Sanger sequences were aligned using ClustalW on BioEdit (ver. 7.2.5) [6]. The sequences were submitted to the NCBI Gen-Bank and accession numbers allocated as presented in Table 1. Genetic diversity indices such as numbers of nucleotide, number of monomorphic/invariable sites, parsimony information sites, total number of mutations, number of haplotypes, haplotype (gene) diversity, nucleotide diversity and average number of nucleotide differences k(i), and codon usage bias were estimated using DnaSP 6.0 [7]. We also estimated genetic differentiation parameters among the three partitioned populations of *L. taraxacifolia*. The codon usage indices and amino acid residues were estimated using CodonW as implemented on a public Galaxy server [8]. The phylogenetic tree to reveal the evolutionary relationship among the studied accessions was constructed using the UPGMA method [9].

## Limitations

We identify two limitations:

1. Difficulty in collecting many accessions from the wild because *L. taraxacifolia* is inching toward extinction.
2. Limitation of the partial rbcL to satisfactorily discriminate the *L. taraxacifolia* accessions as expected. Nevertheless, the partial rbcL deciphered the intra-specific genetic diversity and phylogenetic relationship of the species even though it is a segment of the gene that only contains a portion (large subunit) of the entire coding sequence. The partial rbcL used in this analysis is highly conserved with a similarity of 100 % among and between the same species. However, we recommend further genetic diversity analysis of *L. taraxacifolia* using diversity array technology sequence (DArTseq) which provides comprehensive genome coverage without requiring DNA sequence information like rbcL marker.

## Ethics statement

The data of the sample collection as shown in Table 1 was obtained through field trips and no informed consent is required. No part of the data was obtained from any Social Media platform.

## Data availability

Partial rbcL gene sequences of 15 *Launaea taraxacifolia* (Original data) (GenBank NCBI (Popset)).

## CRediT Author Statement

Abiodun Sunday Oyelakin: Conceptualization, Supervision, Validation, Original draft writing; Jacob Olagbenro Popoola: Review manuscript; Data curation and analysis; Manuscript submission; Babalola Favour Olanrewaju: Accession collection; Formal analysis, Methodology, Submission of Sequences on GenBank; Bamiro Taiwo Adebowale: Accession collection, Methodology, review of manuscript; Oladapo Gloria: Accession collection, review, and editing of manuscript; Oluwatuyi Victor Olamide: Accession collection, Experimentation, Oke Oluwatobi Faith: Accession collection; Data analysis; Sobayo Al-fuad: Accession collection, Experimentation, Methodology.

## Data Availability

Launaea taraxacifolia ribulose-1,5-bisphosphate carboxylase/oxygenase large subunit (rbcL) gene, partial cds; chloroplast. (Original data).
